# Activation of the Keap1/Nrf2 stress response pathway in autophagic vacuolar myopathies

**DOI:** 10.1186/s40478-016-0384-6

**Published:** 2016-10-31

**Authors:** Steve Duleh, Xianhong Wang, Allison Komirenko, Marta Margeta

**Affiliations:** 1 School of Medicine, University of California, San Francisco, CA USA; 2 Department of Pathology, University of California, San Francisco, CA USA; 3 School of Pharmacy, University of California, San Francisco, CA USA

**Keywords:** Autophagy, Stress response signaling, Keap1, Nrf2, Hydroxychloroquine, Colchicine, Toxic myopathy, Vacuolar myopathy, Inclusion body myositis

## Abstract

Nrf2 (nuclear factor [erythroid-d﻿erived 2]-like 2﻿;﻿ the transcriptional master regulator of the antioxidant stress response) is regulated through interaction with its cytoplasmic inhibitor Keap1 (Kelch-like ECH-associated protein 1), which under basal conditions targets Nrf2 for proteasomal degradation. Sequestosome 1﻿ ﻿(SQSTM1)/p62–a multifunctional adapter protein that accumulates following autophagy inhibition and can serve as a diagnostic marker for human autophagic vacuolar myopathies (AVMs)–was recently shown to compete with Nrf2 for Keap1 binding, resulting in activation of the Nrf2 pathway. In this study, we used 55 human muscle biopsies divided into five groups [normal control, hydroxychloroquine- or colchicine-treated non-AVM control, hydroxychloroquine- or colchicine-induced toxic AVM, polymyositis, and inclusion body myositis (IBM)] to evaluate whether Keap1-SQSTM1 interaction led to increased Nrf2 signaling in human AVMs. In toxic AVMs and IBM, but not in control muscle groups or polymyositis, Keap1 antibody labeled sarcoplasmic protein aggregates that can be used as an alternate diagnostic marker for both AVM types; these Keap1-positive aggregates were co-labeled with the antibody against SQSTM1 but not with the antibody against autophagosome marker LC3 (microtubule-associated protein 1 light chain 3). In human AVM muscle, sequestration of Keap1 into the SQSTM1-positive protein aggregates was accompanied by an increase in mRNA and protein levels of Nrf2 target genes; similarly, treatment of differentiated C2C12 myotubes with autophagy inhibitor chloroquine led to an increase in the nuclear Nrf2 protein level and an increase in expression of the Nrf2-regulated genes. Taken together, our findings demonstrate that Nrf2 signaling is upregulated in autophagic muscle disorders and raise the possibility that autophagy disruption in skeletal muscle leads to dysregulation of cellular redox homeostasis.

**Electronic supplementary material:**

The online version of this article (doi:10.1186/s40478-016-0384-6) contains supplementary material, which is available to authorized users.

## Introduction

Macroautophagy (hereafter referred to as autophagy) is a catabolic pathway that contributes to cellular homeostasis by mediating routine protein and organelle turnover through lysosomal degradation [1, 2]. An early step in autophagy induction is lipidation of the microtubule-associated protein 1 light chain 3 (LC3, a mammalian orthologue of yeast ATG8); LC3-II (the lipidated form of LC3) associates with the newly generated autophagosome membrane and is thus commonly used as a marker of autophagosome formation [3]. LC3-II also binds multifunctional adapter protein SQSTM1/p62, which through this interaction targets ubiquitinated protein aggregates for lysosomal degradation [4]. Because SQSTM1 is selectively degraded through autophagy, its accumulation can be used as another measure of disrupted autophagic flux [3].

Autophagic disruption is a hallmark of several inherited skeletal myopathies including X-linked myopathy with excessive autophagy and infantile autophagic vacuolar myopathy [5, 6]; these disorders are characterized by defects in lysosomal degradation that lead to secondary accumulation of autophagic vacuoles [5]. Autophagic vacuolar myopathies (AVMs) can also develop as a toxic side effect of treatment with autophagy inhibiting drugs such as chloroquine (CQ; used for treatment of malaria), hydroxychloroquine (HCQ; used for treatment of rheumatologic disorders), and colchicine (used for treatment of gout) [7–9]. CQ and its derivative HCQ are thought to inhibit autophagy through lysosome alkalinization, which interferes with function of the pH-sensitive lysosomal enzymes [10]; in contrast, colchicine disrupts microtubule cytoskeleton, thus inhibiting autophagosome-lysosome fusion (which requires translocation of autophagosomes along microtubules) [9]. Interestingly, all AVMs clinically present with muscular weakness but only a minority of AVM muscle biopsies show muscle fiber degeneration (a classic feature of both inherited and toxic myopathies); thus, the causal mechanism linking autophagy impairment with muscle weakness remains poorly understood.

Skeletal muscle from patients with drug-induced/toxic AVMs exhibits accumulation of both LC3-II and SQSTM1, either in the fiber center or in association with rimmed vacuoles (sarcoplasmic vacuoles that on hematoxylin & eosin or trichrome stains show a basophilic rim) [11]. Intriguingly, rimmed vacuoles are also a hallmark feature of inclusion body myositis (IBM), a common and currently untreatable myopathy of the elderly that is characterized by both inflammatory and degenerative features as well as evidence of abnormal protein aggregation [12–14]. As with muscle from patients with toxic AVMs, skeletal muscle from IBM patients shows abundant LC3-II and SQSTM1 sarcoplasmic puncta that can be used to morphologically distinguish IBM from polymyositis (PM), a histologically similar inflammatory myopathy that generally occurs in younger patients and responds well to immunosuppressive therapy [15]. While IBM pathogenesis is not well understood, the presence of autophagy defects in this disorder suggests a possible mechanistic link with inherited and toxic AVMs.

The Keap1/Nrf2 stress response pathway controls redox homeostasis in eukaryotic cells. Nrf2 is a Cap’n’Collar basic leucine zipper transcription factor that regulates expression of a battery of antioxidant enzymes, chaperones, and other stress response proteins [16, 17]. At baseline, the Nrf2 pathway is negatively regulated by Keap1, a cytoplasmic regulatory protein that targets Nrf2 for proteasomal degradation through interaction with the Cullin-E3 ubiquitin ligase [17, 18]. Canonical activation of Nrf2 signaling–for example, during oxidative stress or in response to various phytochemicals–is mediated by a conformational change in Keap1 that leads to disruption of the Keap1/Nrf2 protein complex, allowing Nrf2 to evade degradation, translocate to the nucleus, and initiate transcription of genes with Antioxidant Response Element (ARE) in their promoter [17]. Recent work has demonstrated that Nrf2 signaling can also be activated through a non-canonical, SQSTM1-mediated pathway: because SQSTM1 competes with Nrf2 for Keap1 binding, SQSTM1 accumulation disrupts Nrf2 degradation, ultimately leading to increase in cytoplasmic and nuclear Nrf2 levels and elevated transcription of the Nrf2-regulated genes [19–22]. Canonical Nrf2 activation is generally cytoprotective [17], but persistent Nrf2 activation caused by autophagy disruption has been linked to liver toxicity and development of hepatocellular carcinoma [19, 21–23]. In the current study, we investigated whether autophagy inhibition in AVMs leads to abnormal sequestration of Keap1 protein and subsequent activation of the Keap1/Nrf2 stress response pathway.

## Materials and methods

### Participants

In the current study, we used muscle biopsies from two previously described human subject cohorts; detailed criteria for subject selection and classification are described in the original publications [11, 15]. Briefly, the toxic AVM cohort included three groups of human subjects [11]: (i) subjects with HCQ- or colchicine-induced AVM (the toxic AVM group), (ii) subjects with a history of HCQ or colchicine treatment but no evidence of AVM on muscle biopsy (the drug-treated control group), and (iii) subjects with normal muscle biopsy findings and no history of HCQ or colchicine use (the normal control group). Classification of drug-treated subjects into the control and toxic AVM groups was based on a blind review of 10 or more electron micrographs by two experienced neuromuscular pathologists; ultrastructural identification of at least 15 definitive autophagic vacuoles in the image set was required for specimen classification in the toxic AVM group [11]. The IBM cohort included two of the four groups from the original study [15]: (i) the PM group and (ii) the IBM group. Subject classification into the PM and IBM groups was based on a blind review of archival microscopic slides by two experienced neuromuscular pathologists using the pathologic criteria summarized in Additional file 1: Table S1; because approximately two thirds of muscle biopsies came from outside referring institutions and were accompanied by limited clinical information, the clinical features were not incorporated into the diagnostic criteria except to exclude subjects with systemic disease (such as HIV infection or an autoimmune disorder) [15]. [When compared to the clinicopathologic criteria used to diagnose IBM in clinical practice, the morphologic criteria used to diagnose IBM in biopsy specimens are equally specific although less sensitive [14, 24]; thus, they remain a suitable choice for subject selection and classification in research studies that investigate molecular and cell biological aspects of IBM using archival biopsy material.] Several specimens used in our earlier work were no longer available because of tissue exhaustion; to maintain group size, new subjects were added to the drug-treated control, toxic AVM, and IBM groups using the same selection and classification criteria [11, 15]. The human subject data is summarized in Table 1; newly added subjects are designated by asterisks. Given that group assignment was based solely on the history of HCQ or colchicine use (for the toxic AVM cohort) and morphologic criteria (for both cohorts), no attempt was made to match participants by age, sex, or other demographic variables. Study design was reviewed and approved by the University of California San Francisco Committee on Human Research (CHR). Given the non-invasive nature of the study and a minimal potential for harm to the study participants, the informed consent requirement was waived by the CHR. No individually identifiable patient data is presented in the current report.

**Table 1:** Study subject characteristics

Subject ID	Age	Sex	Drug	Biopsy diagnosis	LC3 (% positive fibers)	SQSTM1 (% positive fibers)	Keap1 (% positive fibers)
Normal control group							
1	52	F	None	Normal	0.0	0.0	0.0
2	67	F	None	Normal	0.0	0.0	0.0
3	83	F	None	Normal	0.0	0.0	0.0
4	56	F	None	Normal	0.0	0.0	0.3
5	53	M	None	Normal	0.0	0.0	0.0
6	57	M	None	Normal	0.0	1.0	0.2
7	60	M	None	Normal	0.0	0.0	0.0
8	64	F	None	Normal	0.0	0.0	ND
9	48	M	None	Normal	0.0	0.0	0.0
10	32	M	None	Normal	0.0	0.0	0.0
Drug-treated control group							
11	32	F	HCQ	Necrotizing myopathy	13.0	10.5	4.5
12	58	F	HCQ	Inflammatory myopathy	2.5	4.3	2.0
13	33	M	HCQ	Normal	0.3	0.2	0.5
14	65	F	HCQ	Neurogenic changes	4.0	2.0	1.0
15	44	F	Colchicine	Neurogenic changes	1.8	1.5	1.0
16	47	F	HCQ	Normal	0.8	0.3	1.0
17^a^	60	F	HCQ	Polymyositis; neurogenic changes	0.5	2.0	2.0
18^a^	59	F	HCQ	Normal	4.3	2.3	3.8
19^a^	31	F	HCQ	Inflammatory myopathy	0.8	0.5	2.0
Toxic autophagic vacuolar myopathy group							
20	73	M	Colchicine	Toxic AVM	64.0	86.0	72.0
21	58	M	Colchicine	Toxic AVM	22.5	14.8	8.0
22	81	F	Colchicine	Toxic AVM	78.0	83.5	87.0
23	84	M	Colchicine	Toxic AVM	58.5	65.0	60.0
24	80	M	Colchicine	Toxic AVM	13.0	21.0	34.0
25	72	F	HCQ	Toxic AVM	56.5	53.0	45.5
26	33	F	HCQ	Toxic AVM	18.0	12.5	13.5
27	79	M	Colchicine	Toxic AVM	79.0	79.5	49.0
28	79	M	Colchicine	Toxic AVM	95.0	93.0	78.0
29	29	M	HCQ	Toxic AVM	12.5	25.5	31.5
30^a^	62	F	Colchicine	Toxic AVM	21.8	27.3	37.3
31^a^	66	M	Colchicine	Toxic AVM	17.5	18.3	10.5
32^a^	24	F	HCQ	Toxic AVM	10.5	12.5	14.0
Polymyositis group							
33	59	M	None	Polymyositis	5.7	5.5	5.0
34	85	M	None	Polymyositis	6.0	10.0	6.0
35	51	F	None	Polymyositis	4.0	0.3	0.5
36	65	M	None	Polymyositis	2.5	3.7	1.5
37	47	M	None	Polymyositis	1.7	11.0	9.0
38	42	M	None	Polymyositis	11.3	10.2	3.0
39	66	F	None	Polymyositis	3.8	4.0	0.5
40	59	M	None	Polymyositis	6.7	8.3	5.5
41	53	M	None	Polymyositis	2.7	19.2	12.5
42	43	M	None	Polymyositis	2.7	4.0	1.5
43	50	M	None	Polymyositis	2.0	2.8	0.5
Inclusion body myositis group							
44	64	F	None	IBM	31.0	18.5	24.0
45	57	F	None	IBM	24.0	20.0	34.0
46	62	M	None	IBM	29.5	59.5	48.5
47	77	M	None	IBM	15.5	16.5	15.5
48	74	F	None	IBM	16.0	9.0	7.0
49	73	F	None	IBM	34.0	32.0	24.0
50	69	F	None	IBM	48.5	43.5	20.5
51	75	M	None	IBM	11.0	22.5	20.0
52	59	M	None	IBM	33.5	36.0	40.0
53	66	M	None	IBM	9.0	15.0	6.5
54	76	M	None	IBM	32.5	12.0	27.5
55^b^	80	M	None	IBM	9.5	13.5	11.8

*ND* Not determined (FFPE tissue was exhausted, but the subject was retained in the cohort because frozen tissue for qRT-PCR analysis was available)

^a^Subject added to the cohort after the publication of reference [11]

^b^Subject added to the cohort after the publication of reference [15]

Note: This table includes only the information regarding treatment with autophagy inhibiting drugs (HCQ and colchicine), not the full list of patient medications

### Immunohistochemistry

Immunoperoxidase staining of FFPE tissue was performed using the Ventana Benchmark XT automated slide preparation system at the UCSF Brain Tumor SPORE Tissue Core. Briefly, tissue sections (4–5 μm thickness) were deparaffinized (EZ-Prep, Ventana Medical Systems, at 75 °C), incubated in antigen retrieval buffer (Cell Conditioning 1, Ventana Medical Systems) at 95–100 °C, and then incubated with primary antibodies [LC3 (mouse monoclonal antibody, clone 5F10, Nanotools; 1:100 dilution), SQSTM1 (guinea pig polyclonal antibody, catalog number GP62-C, Progen Biotechnik; 1:100 dilution), or Keap1 (rabbit polyclonal antibody, catalog number 10503-2-AP, ProteinTech; 1∶500 dilution unless indicated otherwise)] for 2 h at room temperature. Staining was developed using the UltraView Universal DAB detection system (Ventana Medical Systems) followed by hematoxylin counterstain.

### Quantification of immunopositive fibers

Quantification was performed on immunostained sections of FFPE material using a bright field light microscope, with the investigator blinded to the group assignment of each subject. Each slide was first viewed at both low (10–20×) and high power (40×) to qualitatively assess the distribution of immunostaining and was then divided into 4 quadrants. In samples with uniform staining, 50 fibers were counted from each quadrant (by counting adjacent fibers from 2–3 randomly selected high power fields) for a total of 200 fibers per slide; in samples with scarce or non-homogenous staining, 150 fibers in each quadrant (adjacent fibers from 5–6 high power fields per quadrant; 600 fibers total) were counted to decrease the sampling error. A fiber was considered positive if it contained frequent Keap1-positive coarse sarcoplasmic puncta (>25 on a cross section or >50 on a longitudinal section) and/or at least one large Keap1-positive inclusion (protein aggregate). The number of positive fibers was divided by the total number of fibers counted to determine the percentage of positive fibers.

### Immunofluorescence

Immunofluorescence staining was performed on a subset of specimens from the normal control, toxic AVM and IBM groups (3–5 specimens per group). 8 μm thick frozen sections were mounted on Superfrost Plus Microscope Slides (Fisherbrand), fixed with 4 % paraformaldehyde in PBS for 30 min, and then permeabilized with 0.5 % NP40 in PBS for 10 min (all at room temperature). After fixation and permeabilization, sections were blocked for 1 h at room temperature in the blocking buffer (5 % goat serum and 0.2 % Triton X-100 in PBS), incubated with rabbit polyclonal anti-Keap1 antibody (ProteinTech, 10503-2-AP; 1:100 in blocking buffer) overnight at 4 °C, and then incubated with the secondary antibody (Alexa Fluor 488-conjugated goat anti-rabbit IgG, Life Technologies, A11070; 1:200 in blocking buffer) for 1 h at room temperature. Sections were then again blocked for 1 h at room temperature, incubated with guinea pig polyclonal anti-SQSTM1 antibody (Progen Biotechnik, GP62-C; 1:100 in blocking buffer) or mouse monoclonal anti-LC3 antibody (Nanotools, clone 5F10; 1:50 in blocking buffer) overnight at 4 °C, and finally incubated with the appropriate secondary antibody [Alexa Fluor 594-conjugated goat anti-guinea pig (Life Technologies, A11076) or goat anti-mouse IgG (Life Technologies, A11005); 1:200 in blocking buffer)] for 1 h at room temperature. Nuclear DAPI stain (4′,6-diamidino-2-phenylindole, Sigma; 1 μg/ml) was used as a counterstain. All washes were done with 0.2 % Triton X-100 in PBS. To generate negative control slides for imaging optimization, sections were processed in the same way except that both primary and secondary antibodies were omitted from the blocking buffer.

### Imaging

A BX41 bright field light microscope (Olympus) equipped with a DP72 digital camera (Olympus) and cellSens entry 1.4 software (Olympus) was used for bright field image acquisition. Fluorescence images were acquired with a spinning disk confocal microscope (Yokogawa) using Micro Manager acquisition software (v1.4.22). To prevent imaging of baseline lipofuscin autofluorescence, acquisition software settings were calibrated using a no-antibody negative control slide for each case. Images were acquired as a Z-series with a 1 μm step increment, with three or more high power fields imaged for each slide. Image processing was performed using free ImageJ software (v1.50i), with final editing done using Adobe Photoshop CC (2015.1.2 release).

### Cell culture and CQ treatment

C2C12 cells (a gift from Dr. Jason Pomerantz, UCSF) were maintained in the growth medium [high glucose DMEM (4.5 g/L glucose; UCSF Cell Culture Facility) supplemented with 10 % FBS (Gibco; 16000-044) and 10 μg/mL penicillin/10 U/mL streptomycin (UCSF Cell Culture Facility)]. After reaching approximately 80 % confluence, cells were moved to the differentiation medium [high glucose DMEM supplemented with 2 % horse serum (HyClone, SH30074.03), 10 μg/mL penicillin, and 10 U/mL streptomycin]. Experiments were performed after myoblasts fused into myotubes (3–6 days after transfer to the differentiation medium). CQ was purchased from InvivoGen (catalog number tlrl-chq) in a powder form, with 10 mM stock solution prepared in water and stored in small aliquots at -20 °C, and was applied in the differentiation medium; concentration and treatment duration are indicated in the figure or figure legend.

### Immunoblotting

Snap-frozen human muscle tissue was homogenized at 4 °C in RIPA lysis buffer (Invitrogen) on ice in the presence of protease and lysosome inhibitors; samples where then sonicated on ice at 2 W using Fischer Scientific 60 Sonic Dismembranator to generate crude homogenates, which were cleared by subsequent centrifugation at 14,000 rpm for 15 min at 4 °C. Samples were boiled in the SDS sample buffer (Invitrogen) and resolved by 4–12 % Bis-Tris NuPAGE gels (Invitrogen); 15 μg of total protein was loaded per lane. Nuclear and cytoplasmic protein-enriched fractions from C2C12 cells were prepared using the NE-PER kit (Pierce) according to the manufacturer’s instructions. After solubilization with LDS sample buffer (Invitrogen), samples were heated for 10 min at 70 °C and then electrophoretically resolved with 4–12 % NuPAGE Bis-Tris gels; 40 μg of protein was loaded per lane. For both preparations, gels were electroblotted to nitrocellulose membranes. 5 % (wt/vol) BSA in TBS (150 mM NaCl, 20 mM TrisCl; pH 7.4) supplemented with 0.1 % Tween (TBST) was used for dilution of primary antibodies, while 5 % (wt/vol) nonfat dried milk in TBST was used for blocking and dilution of secondary antibodies. Membranes were blocked for 1 h at room temperature; incubated with primary antibodies for 2–3 h at room temperature or overnight at 4 °C; washed three times for 30 min at room temperature; incubated with corresponding secondary antibody for 1 h at room temperature; and washed for at least 30 min at room temperature. Following a final wash in TBS with 0.1 % Tween for 10 min at room temperature, protein–antibody complexes were detected using an ECL chemiluminescent kit (Pierce Biotechnology) and Premium Autoradiography Film (Denville Scientific) with a Konica SRX-101A film developer. Primary antibodies included mouse monoclonal anti-Nqo1 (clone A180; Abcam, ab28947; 1:500); mouse monoclonal anti-Hmox1 (clone HO-1-1; Abcam, ab13248; 1:250); rabbit polyclonal anti-Nrf2 (Santa Cruz Biotechnology, H300; 1:400); goat polyclonal anti-Lamin B (Santa Cruz Biotechnology, C20; 1:200); mouse monoclonal anti-Keap1 (Proteintech, 60027-1-Ig; 1:100); rabbit polyclonal anti-LC3 (Novus Biological, NB100-2220SS; 1:250); guinea pig polyclonal anti-SQSTM1 (GP62-C, Progen Biotechnik; 1:1,000); mouse monoclonal anti-GAPDH (clone 6C5, Millipore, MAB374; 1:2,000). Secondary antibodies included goat HRP-conjugated anti-rabbit secondary H + L IgG antibody (Jackson ImmunoResearch; 1:10,000); bovine HRP-conjugated anti-goat secondary H + L IgG antibody (Santa Cruz Biotechnology; 1:10,000); goat HRP-conjugated anti-mouse secondary H + L IgG antibody (Jackson ImmunoResearch; 1:5,000); and goat HRP-conjugated anti-guinea pig IgG (Santa Cruz Biotechnology; 1:5,000).

### Quantitative RT-PCR

Total RNA was isolated from human muscle samples using RNeasy Fibrous Tissue Mini Kit and from C2C12 cells using RNeasy Mini Kit (both from Qiagen), with contaminating genomic DNA removed during isolation via an on-column DNase digestion step; RNA concentration was measured using Nanodrop 1000. mRNA was reverse-transcribed to cDNA using Cloned AMV First-Strand cDNA Synthesis Kit (Invitrogen) according to the manufacturer’s instructions. Quantitative PCR reactions were carried out on CFX96 Real-Time System (Bio-Rad) using a 10-ng sample in a 20-μL, 98-well format. SsoAdvanced Universal Probes Supermix was purchased from Bio-Rad, and TaqMan PCR primer and probe sets were purchased from Applied Biosystems for all mRNAs measured; GAPDH was used for normalization. For human samples, the following probe sets were used: Nqo1, Hs02512143_s1; Gclc, Hs00155249; Gclm, Hs00157694_m1; Hmox1, Hs01110250_m1; Gsr, Hs00167317_m1; G6pdx, Hs00166169_m1; GAPDH, Hs02758991_g1. The common reference cDNA (used to prepare the standard curve for all reactions) was Human Brain Total RNA (Applied Biosystems). For mouse C2C12 cell samples, the following probe sets were used: Nqo1, Mm01253561_m1; Gclc, Mm00802655_m1; Gclm, Mm00514996_m1; Hmox1, Mm00516005_m1; Gsr, Mm00439154_m1; G6pdx, Mm00656735_g1; GAPDH: Mm99999915_g1. The common reference cDNA for this sample set was generated by mixing total RNA from several untreated culture batches. Two technical replicates for each sample were run on one plate, with replicate plates ran for a subset of experiments to confirm accuracy.

### Statistical analysis

Statistical analyses were performed with GraphPad Prism 6.0 statistical software; the specific test used for each experiment was chosen based on the experimental design and is specified in the main text or figure legends. All tests were two-tailed, with *p* < 0.05 considered statistically significant.

## Results

### Keap1 immunohistochemistry in toxic AVMs

**Fig. 1:**
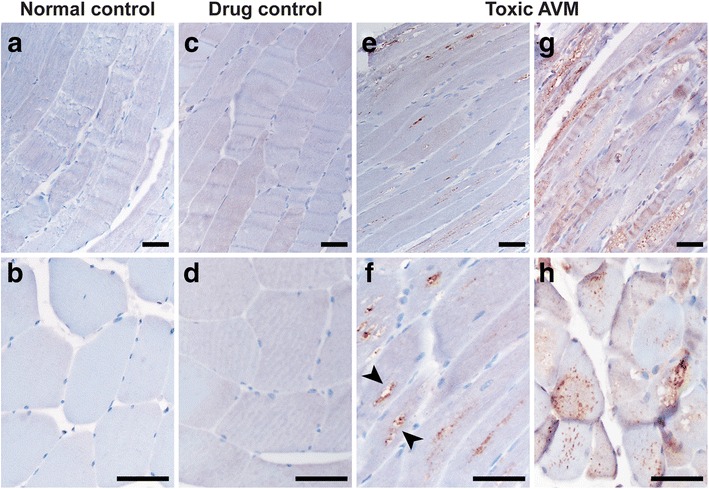
Keap1 immunohistochemistry labels coarse sarcoplasmic puncta in toxic AVM muscle. **a**-**d**. Faint diffuse sarcoplasmic staining is seen in muscle samples from a normal control subject (#10; **a**-**b**) and a drug-treated control subject (#17; **c**-**d**). **e**-**h**. Frequent fibers with the Keap1-positive coarse sarcoplasmic puncta are observed in muscle specimens from subjects in the toxic AVM group. The Keap1-positive puncta were often seen in the center of the fiber (aligned with the longitudinal fiber axis; **e**) or adjacent to/within sarcoplasmic vacuoles (*arrowheads*, **f**); in other cases, the puncta were seen throughout the fiber sarcoplasm (**g**-**h**). (**e**-**f**, HCQ-treated subject #25; **g-h**, colchicine-treated subject #22). Scale bars, 50 μM

To determine whether autophagy impairment alters Keap1 subcellular localization in the human skeletal muscle, immunohistochemistry for Keap1 was performed on FFPE tissue from human subjects with no detectable muscle disease (the normal control group), human subjects treated with colchicine or HCQ but no evidence of AVM (the drug-treated control group), and human subjects with colchicine- or HCQ-induced AVM (the toxic AVM group). Using a high dilution of the Keap1 antibody, little to no staining was observed in muscle specimens from the normal and drug-treated control groups (Fig. 1a-d). In contrast, muscle samples from the toxic AVM group showed coarse Keap1-positive sarcoplasmic puncta that often localized to the area of myofibrillary disorganization and/or vacuolization in the fiber center (Fig. 1e-h); this coarsely punctate staining pattern was never observed in the normal muscle and was only rarely seen in the muscle from the drug-treated control subjects. [With lower antibody dilutions, the control muscle showed a checkerboard pattern of diffuse sarcoplasmic Keap1 staining, raising a possibility that Keap1 protein is expressed at different levels by fast and slow twitch muscle fibers (Additional file 2: Figure S1A); future work will be required to fully evaluate this possibility. Under these staining conditions, it was still possible to observe Keap1 sequestration into coarse puncta in specimens from the toxic AVM group (Additional file 2: Figure S1B-C), but this disease-specific staining pattern was more difficult to distinguish from the normal background Keap1 staining; thus, a high antibody dilution was used for subsequent quantification of fibers with Keap1-positive sarcoplasmic aggregates.

**Fig. 2:**
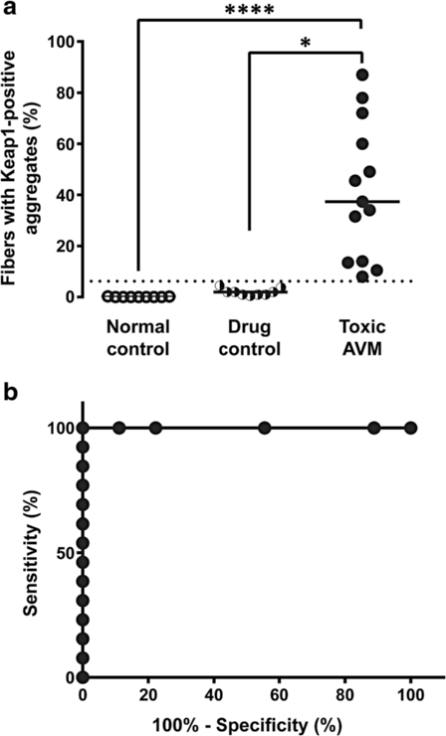
Quantification of fibers with Keap1-positive coarse sarcoplasmic puncta differentiates toxic AVM from control muscle specimens. **a**. The percentage of fibers with Keap1-positive coarse puncta was significantly higher in the toxic AVM group (*solid circles*) than in the normal control group (*open circles*) or the drug-treated control group (*semi-solid circles*). Each study subject is represented with a symbol; solid lines indicate group medians. The dashed line indicates a cutoff value with a 100 % sensitivity and 100 % specificity (6.25 %), as determined by ROC analysis. (****, *p* < 0.0001; *, *p* < 0.05; Kruskal-Wallis ANOVA on ranks). **b**. ROC analysis indicates that among colchicine- or HCQ-treated subjects, quantification of muscle fibers with Keap1-positive coarse sarcoplasmic puncta successfully differentiates AVM from control cases (area under the curve = 1.00; *p* < 0.0001)

To statistically compare the degree of Keap1 sequestration into protein aggregates among the three experimental groups, we quantified the percentage of muscle fibers with Keap1-positive coarse puncta in FFPE sections; individual subject data are shown in Table 1. The percentage of fibers with Keap1-positive protein aggregates was significantly higher in the toxic AVM group (median 37.3 %, SD 26.7 %) than in the normal control group (median 0.0 %, SD 0.1 %; *p* < 0.001) or the drug-treated control group (median 2.0 %, SD 1.4 %; *p* < 0.05) (Fig. 2a; Kruskal-Wallis one-way ANOVA on ranks). To assess the value of Keap1 immunopositivity as a diagnostic marker of toxic AVMs, we performed ROC (receiver-operator characteristic) curve analysis using the data from the two drug-treated groups (Fig. 2b). ROC analysis showed that Keap1 immunohistochemistry can effectively distinguish the toxic AVM from drug-treated control specimens (area under ROC curve = 1.00; *p* < 0.0001), with 100 % sensitivity and 100 % specificity for toxic AVM using a threshold value of 6.25 % fibers with Keap1-positive puncta (a result comparable to sensitivity and specificity of LC3 or SQSTM1 immunohistochemistries for diagnosis of the same condition [11]).

### Keap1 immunohistochemistry in PM and IBM

**Fig. 3:**
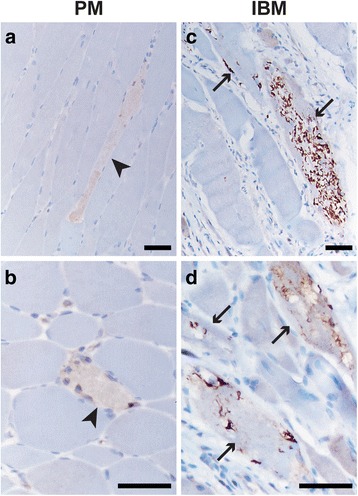
Keap1 immunohistochemistry labels large sarcoplasmic inclusions in IBM muscle. **a**-**b**. In a representative PM specimen (subject #38), there is an increase in diffuse sarcoplasmic Keap1 staining in necrotic fibers (*arrowheads*), but no Keap1-positive sarcoplasmic puncta or inclusions are detected. **c**-**d**. In a representative IBM sample (subject #52), the Keap1-positive sarcoplasmic inclusions are observed in multiple fibers (*arrows*). Scale bars, 50 μM

**Fig. 4:**
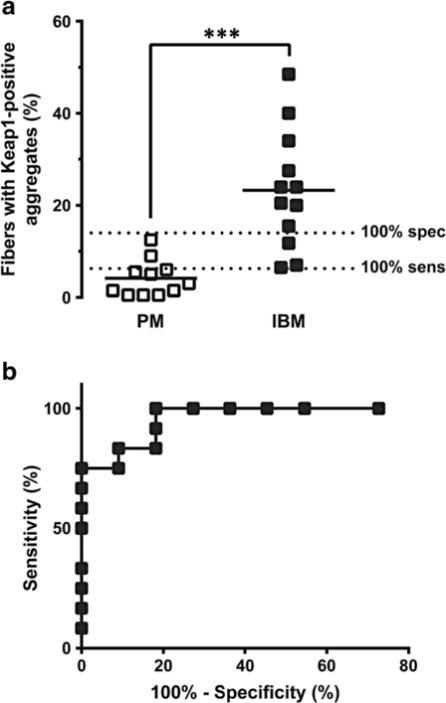
Quantification of fibers with Keap1-positive sarcoplasmic inclusions differentiates IBM from PM specimens. **a**. The percentage of fibers with the Keap1-positive sarcoplasmic puncta and/or inclusions was significantly higher in the IBM group (*solid squares*) than in the PM group (*open squares*). Each study subject is represented with a symbol; solid lines indicate group medians. Dashed lines indicate the cutoff values for 100 % sensitivity (6.25 %) and 100 % specificity (14.00 %), as determined by ROC analysis. (***, *p* < 0.001; unpaired *t*-test with Welch’s correction for unequal variances). **b**. ROC analysis indicates that quantification of fibers with the Keap1-positive sarcoplasmic aggregates successfully differentiates IBM from PM cases (area under the curve = 0.96; *p* < 0.0001)

Colchicine and CQ/HCQ-induced toxic AVMs represent a pure form of autophagic impairment, but are relatively rare. To expand our findings to a broader spectrum of human AVMs, we performed Keap1 immunohistochemistry on FFPE tissue from subjects with IBM, a treatment-resistant inflammatory myopathy that shows evidence of autophagy dysregulation [15, 25, 26]. As a control group for this part of the study, we used muscle biopsies from subjects with PM, a treatable inflammatory myopathy that shares many histopathologic and immunologic features with IBM but lacks both protein aggregates and evidence of impaired autophagy [15, 26]. When stained with a low concentration of the anti-Keap1 antibody (as described in the section on toxic AVMs), PM muscle biopsies generally showed little to no sarcoplasmic staining except in necrotic fibers and inflammatory cells (Fig. 3a-b); in some PM specimens, rare fibers showed small Keap1 puncta, but labeling of large protein aggregates was not observed. In IBM specimens, in contrast, Keap1 antibody labeled many large sarcoplasmic protein aggregates in addition to coarse sarcoplasmic puncta usually present in the same fibers (Fig. 3c-d). Keap1-positive sarcoplasmic aggregates were seen in a significantly higher percentage of muscle fibers in specimens from the IBM group (median 22.5 %, SD 12.8 %) than from the PM group (median 3.0 %, SD 3.9 %) (Fig. 4a; *p* = 0.0003, unpaired *t*-test with Welch’s correction). ROC analysis of the same data showed that Keap1 immunohistochemistry can effectively distinguish IBM from PM specimens (Fig. 4b; area under the curve = 0.96; *p* < 0.0001), although the trade-off between sensitivity and specificity was larger than for toxic AVMs (Fig. 2b). Specifically, 100 % specificity and 82 % sensitivity for IBM was attained using a threshold value of 14.00 %, while 75 % specificity and 100 % sensitivity for IBM was achieved with a threshold value of 6.25 % fibers with the Keap1-positive protein aggregates; these sensitivity/specificity trade-offs are comparable to those seen with either LC3 or SQSTM1 immunohistochemistry for the same pair of disorders [15]. Thus, Keap1 redistribution from the sarcoplasm to protein aggregates/sarcoplasmic inclusions can be used as an alternate diagnostic marker for both toxic AVMs and IBM.

### Keap1 co-localizes with SQSTM1 in human AVM muscle

**Fig. 5:**
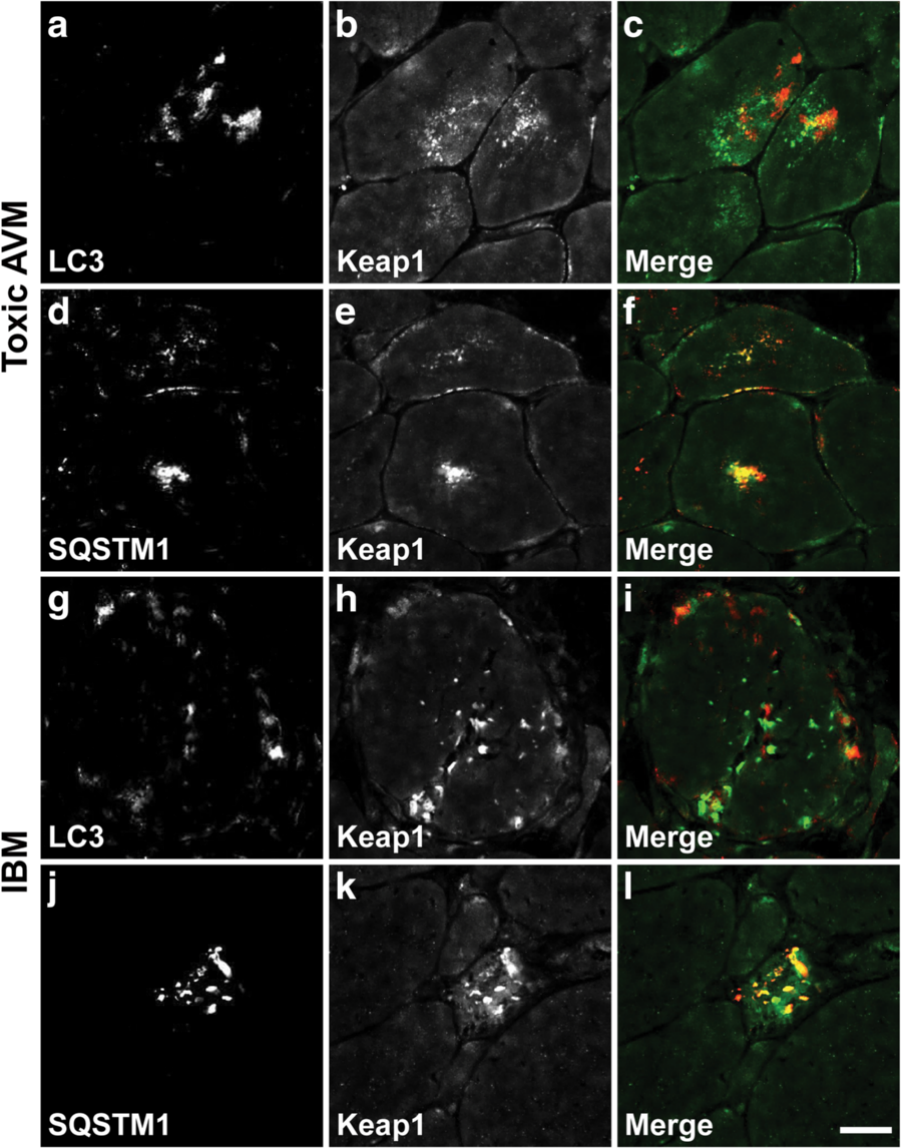
Keap1 co-localizes with SQSTM1-immunopositive sarcoplasmic aggregates in AVM muscle. **a**-**c**. Only focal and minimal co-localization of LC3 (**a**) and Keap1 immunofluorescence (**b**) is seen in a representative toxic AVM sample (colchicine-treated subject #31); merged panel is shown in (**c**) (LC3, red; Keap1, green). **d**-**f**. In the same specimen, there is extensive co-localization of SQSTM1 (**d**) and Keap1 immunofluorescence (**e**); merged panel is shown in (**f**) (SQSTM1, red; Keap1, green). **g**-**i**. Similar to toxic AVM specimens, essentially no co-localization of LC3 (**g**) and Keap1 immunofluorescence (**h**) is seen in a representative IBM muscle biopsy (subject #46); merged panel is shown in (**i**) (LC3, red; Keap1, green). **j**-**l**. In the same specimen, there is extensive co-localization of SQSTM1 (**j**) and Keap1 immunofluorescence (**k**); merged panel is shown in (**l**) (SQSTM1, red; Keap1, green). Scale bar, 25 μm

The Keap1 staining pattern in toxic AVM and IBM muscle biopsies (Figs. 1 and 3) resembles the LC3 and SQSTM1 staining patterns observed in the same specimens [11, 15], raising the possibility that these proteins might be co-localized. To further evaluate this possibility, we performed immunofluorescence staining and confocal imaging on frozen tissue sections from a subset of biopsies; representative images are shown in Fig. 5. Indeed, there was almost perfect co-localization between the Keap1-immunopositive and SQSTM1-immunopositive sarcoplasmic aggregates in both toxic AVM (Fig. 5d-f) and IBM specimens (Fig. 5j-l). In contrast, the anti-LC3 antibody labeled a separate pool of sarcoplasmic aggregates that showed little (if any) co-localization with the Keap1-immunopositive inclusions in either toxic AVM (Fig. 5a-c) or IBM biopsies (Fig. 5g-i). These data suggest that following autophagy inhibition, Keap1 is sequestered into sarcoplasmic protein aggregates through its interaction with accumulated SQSTM1, but does not co-localize with LC3-labeled autophagosomes.

### Autophagy inhibition leads to activation of the Nrf2 signaling pathway

**Fig. 6:**
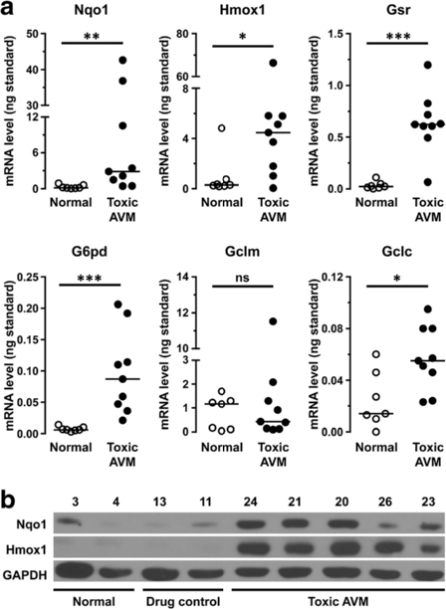
Expression of Nrf2-regulated genes is increased in toxic AVM muscle. **a**. mRNA levels of the Nrf2-regulated genes Nqo1, Hmox1, Gsr, G6pd, and Gclc (but not Gclm) were significantly higher in the toxic AVM group (*solid circles*) than in the normal control group (*empty circles*). Each study subject is represented with a symbol; *solid lines* indicate group medians. Data were not normally distributed and were thus analyzed with the Mann-Whitney non-parametric test; *, *p* < 0.05; **, *p* < 0.01; ***, *p* < 0.001. **b**. In subjects from the toxic AVM group, Nqo1 and Hmox1 protein levels were increased relative to subjects from either normal or drug-treated control groups. Each lane contains a sample from a different study subject, with subject ID numbers indicated on top. GAPDH was used as a loading control

Keap1 sequestration into cytoplasmic aggregates leads to sustained Nrf2 pathway activation in multiple organ systems including the liver [19, 21–23], heart [27, 28], and brain [29]. To establish whether a similar phenomenon occurs in skeletal muscle, we compared mRNA expression level of the Nrf2-regulated antioxidant enzymes between toxic AVM and control muscle specimens (Additional file 3: Table S2 and Fig. 6a). (PM and IBM biopsies were not used for these studies because they contain a large number of inflammatory cells [15], potentially complicating interpretation of biochemical analyses on tissue homogenates that lack cellular resolution.) Five of the six Nrf2-regulated genes studied [NAD(P)H dehydrogenase, quinone 1 (Nqo1), heme oxygenase 1 (Hmox1), glutathione S reductase (Gsr), glucose-6-phosphate dehydrogenase (G6pd), and glutamate-cysteine ligase, catalytic subunit (Gclc)] showed statistically significant mRNA upregulation in the toxic AVM group compared to the control group (Fig. 6a); mRNA level of the sixth gene [glutamate-cysteine ligase, modifier subunit (Gclm)] was not significantly different between the two groups, likely due to a large variation in the baseline Gclm expression and/or a weaker regulation of Gclm by Nrf2. In agreement with the observed gene transcription changes, Hmox1 and Nqo1 protein levels were increased in the skeletal muscle samples from subjects with toxic AVM compared to the skeletal muscle samples from either normal or drug-treated control subjects (Fig. 6b).

**Fig. 7:**
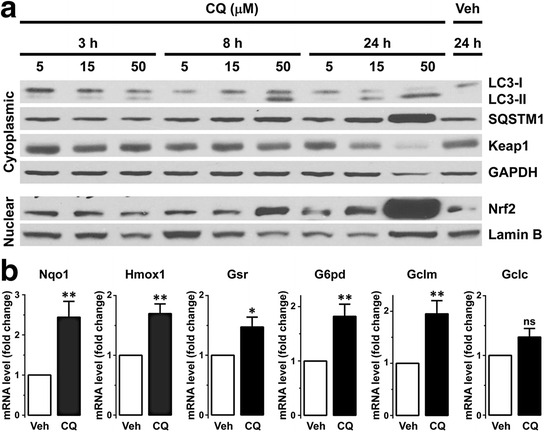
CQ-induced autophagy inhibition leads to Nrf2 pathway activation in C2C12 muscle cells. **a**. Treatment of differentiated C2C12 myotubes with autophagy inhibitor CQ increased the sarcoplasmic level of LC3-II and SQSTM1 proteins in a dose- and time-dependent manner, indicating effective inhibition of the autophagic flux. Autophagy inhibition was accompanied by a slight decrease in the sarcoplasmic level of the soluble Keap1 protein (possibly reflecting its sequestration into insoluble protein aggregates) and an increase in the nuclear level of Nrf2 protein. GAPDH and lamin B were used as loading controls for sarcoplasmic and nuclear protein-enriched fractions, respectively; a representative of 3 independent experiments is shown. **b**. Treatment of C2C12 myotubes with 15 μM CQ for 24 h led to a statistically significant increase in mRNA levels of the Nrf2-regulated genes Nqo1, Hmox1, Gsr, G6pd, and Gclm (but not Gclc). (mean ± SEM; *, *p* < 0.05; **, *p* < 0.01; *n* = 5-8, unpaired *t*-test)

To evaluate whether these findings can be replicated in an in vitro model system, we treated differentiated C2C12 mouse myotubes with autophagy inhibitor CQ. As expected, CQ treatment resulted in accumulation of LC3-II and SQSTM1 proteins in a concentration- and time-dependent manner (Fig. 7a). In parallel with these changes, there was a mild decrease in the soluble sarcoplasmic Keap1 protein level (presumably reflecting its redistribution into insoluble protein aggregates) and an increase in the nuclear Nrf2 protein level in the CQ-treated compared to the vehicle-treated C2C12 myotubes; paralleling autophagy inhibition, an increase in the nuclear Nrf2 level occurred in a concentration-dependent manner, with the active Nrf2 fraction continuing to increase over the 24 h treatment interval (Fig. 7a). To establish whether this increase in the nuclear Nrf2 protein level was accompanied by an increase in transcription of Nrf2 target genes, we examined mRNA levels of the six Nrf2-regulated antioxidant enzymes that were evaluated in human AVM specimens (Fig. 6a). Mimicking the changes that follow autophagy inhibition in vivo, CQ treatment of C2C12 cells in vitro resulted in a statistically significant increase in the mRNA expression level of all but one gene tested (Fig. 7b). Interestingly, autophagy inhibition in murine C2C12 cells led to upregulation of Gclm (modifier subunit of glutamate-cysteine ligase; Fig. 6a), while in human muscle with impaired autophagy there was an increase in expression of Gclc (catalytic subunit of the same enzyme; Fig. 7b); these findings raise the possibility that autophagy inhibition modulates glutamate-cysteine ligase activity through species-specific regulatory mechanisms.

Taken together, our findings indicate that autophagy inhibition in skeletal muscle leads to sequestration of the Nrf2 pathway inhibitor Keap1 into SQSTM1-positive protein aggregates and activation of the Nrf2-mediated stress response pathway.

## Discussion

Non-canonical activation of Nrf2 signaling is mediated through direct interaction of the Nrf2 regulator Keap1 and the adapter protein SQSTM1, which competes with Nrf2 for Keap1 binding [19–21]. Given that SQSTM1 accumulation is a hallmark of human AVMs [11, 15, 30, 31], we performed a retrospective case-control study to evaluate whether SQSTM1-Keap1 interaction leads to activation of the Nrf2 pathway in this category of disorders. Using two previously described cohorts of human AVM subjects and their controls (toxic AVM cohort [11] and IBM cohort [15]), we found that Keap1 is diffusely distributed in the sarcoplasm of normal muscle fibers (Fig. 1 and Additional file 2: Figure S1) but is sequestered into the SQSTM1-positive sarcoplasmic protein aggregates in the AVM muscle (Figs. 1, 3 and 5); in fact, Keap1-positive sarcoplasmic puncta/inclusions can be used as a diagnostic marker for both toxic AVMs (Fig. 2) and IBM (Fig. 4). In addition, we found that Keap1 sequestration was associated with an increase in mRNA and protein expression of the Nrf2-regulated genes (Fig. 6), indicating that the Nrf2 signaling pathway is activated in human AVM muscle; this phenomenon could be replicated in vitro by pharmacologic inhibition of autophagy in cultured murine myotubes (Fig. 7). Together, these findings indicate that autophagy disruption in skeletal muscle results in activation of the Keap1/Nrf2 stress response pathway both in vitro and in vivo.

Historically, pathologic diagnosis of toxic AVMs and IBM required electron microscopy to demonstrate disease-linked ultrastructural abnormalities (autophagosome accumulation in toxic AVMs [11] and presence of 15–18 nm tubulofilamentous inclusions in IBM [14, 32]). More recently, immunohistochemical stains for the autophagosome marker LC3-II and/or protein aggregate marker SQSTM1 have emerged as a more practical alternative: in the appropriate diagnostic setting, these immunohistochemical tests show high sensitivity and specificity for both toxic AVMs and IBM while lowering the cost and improving the turn-around-time compared to ultrastructural analysis [11, 15, 24]. In the current study, we found that immunohistochemical detection of Keap1-positive sarcoplasmic aggregates can be used as an alternate diagnostic test for both toxic AVMs (Figs. 1 and 2) and IBM (Figs. 3 and 4), with sensitivity and specificity that is comparable to LC3-II and SQSTM1 immunohistochemistries [11, 15]. Given that SQSTM1- and Keap1-immunopositive sarcoplasmic aggregates are co-localized in both AVMs (Fig. 5), the close correspondence in diagnostic utility of SQSTM1 and Keap1 immunohistochemistries is not surprising. On the other hand, we found little overlap between LC3-II- and Keap1-immunopositive structures in either AVM disorder (Fig. 5); however, both types of inclusions were generally found in the same muscle fibers, suggesting the explanation for a close correspondence in the diagnostic utility of LC3-II and Keap1 immunohistochemistries. Interestingly, the published research lacks consensus on whether LC3-II co-localizes with SQSTM1/Keap1 protein aggregates. Using cell-free biochemical assays and mammalian cell lines, one study found that Keap1 acts as a competitive antagonist of LC3-SQSTM1 interaction, with increase in Keap1 expression leading to attenuated SQSTM1 degradation via autophagy [33]. In apparent contrast, another study used stably transfected cell lines to show that Keap1 interacts with both LC3-II and SQSTM1 in a stress-inducible manner, facilitating SQSTM1-mediated autophagic clearance of ubiquitin aggregates [34]. Our finding that LC3 does not co-localize with Keap1-immunopositive structures in human AVM muscle (Fig. 5) is more consistent with the former observation; however, additional work will be required to fully elucidate the nature of Keap1/SQSTM1/LC3-II interaction.

What is the effect of chronically elevated Nrf2 signaling on skeletal muscle function and integrity? The canonical activation of the Nrf2 pathway (which is mediated through a transient oxidation-induced change in the Keap1 conformation [35]) is generally beneficial, leading to life span extension in both *C. elegans* and *D. melanogaster* [36, 37]. In agreement with the cytoprotective role of canonical Nrf2 signaling, pharmacologic activation of the Nrf2 pathway is myoprotective in a mouse model of dystrophinopathy [38], while Nrf2 deletion enhances muscle pathology in a mouse model of dysferlinopathy [39], possibly because intact Nrf2 signaling is required for muscle regeneration [40]. In contrast, sustained activation of the Nrf2 pathway generally has negative consequences on organ function and organismal life span; for example, elevated Nrf2 activity promotes tumorigenesis [21, 41] and is associated with a loss of glucose-stimulated insulin secretion in a β cell line [42]. The aberrant effect of sustained Nrf2 activation is particularly evident in the context of impaired autophagy, perhaps because SQSTM1 (an Nrf2-regulated gene) generates a positive feedback amplification loop in the Keap1/Nrf2 pathway that can only be interrupted via its autophagic degradation [33, 43, 44]; in agreement with this notion, chronic activation of Nrf2 signaling in autophagy-deficient livers leads to hepatotoxicity that can be abrogated by deletion of either SQSTM1 or Nrf2 [19, 23]. In the heart, sustained activation of the Nrf2 pathway via Keap1 sequestration into mutant αB-crystallin aggregates leads to reductive stress, a shift of the cellular redox potential to a more reduced state that is both necessary and sufficient for development of fatal cardiac disease [27, 28]. The consequences of chronic Nrf2 activation in skeletal muscle are less well understood; however, mutations in nuclear lamina proteins were recently reported to cause SQSTM1 accumulation, activation of the Nrf2 pathway, and reductive stress in both human and fly muscle [45]. Together, these findings raise the possibility that the aberrant activation of the Nrf2 pathway we observed in the AVM muscle is maladaptive and contributes to AVM pathogenesis; however, further work using cell culture and mouse models will be required to test this hypothesis.

## Conclusion

In summary, we found that SQSTM1-mediated sequestration of Keap1 is associated with chronic activation of the Nrf2 stress response pathway in the AVM muscle, raising the possibility that toxic AVMs and IBM should be added to a growing list of disorders that exhibit dysregulation of cellular redox homeostasis.

## Additional files

Additional file 1: Table S1. Summary of diagnostic criteria used for PM and IBM case classification; adapted from reference [15]. (PDF 11 kb)
Additional file 2: Figure S1. Keap1 immunohistochemistry with a lower antibody dilution. A. When a lower antibody dilution (1:250) was used for Keap1 immunohistochemistry in the normal skeletal muscle (representative subject #1), diffuse sarcoplasmic staining showed a checkerboard distribution, raising a possibility that Keap1 protein is differentially expressed by slow and fast twitch muscle fibers. B-C. Under these experimental conditions, sequestration of Keap1 into sarcoplasmic puncta was still apparent in the toxic AVM muscle (B, HCQ-treated subject #29; C, colchicine-treated subject #20) but was partially obscured by the background Keap1 staining. Scale bar, 20 μm. (PDF 3954 kb)
Additional file 3: Table S2. mRNA expression level for a subset of the Nrf2-regulated genes (individual subject data; summary graphs are shown in Fig. 6a). (PDF 97 kb)

## Abbreviations

ARE: Antioxidant response element
AVM: Autophagic vacuolar myopathy
CQ: Chloroquine
DAPI: 4′,6-diamidino-2-phenylindole
FFPE: Formalin-fixed paraffin-embedded
Gclc: Glutamate-cysteine ligase, catalytic subunit
Gclm: Glutamate-cysteine ligase, modifier subunit
G6pd: Glucose-6-phosphate dehydrogenase
Gsr: Glutathione S reductase
HCQ: Hydroxychloroquine
Hmox1: Heme oxygenase 1
IBM: Inclusion body myositis
Keap1: Kelch-like ECH-associated protein 1
LC3: Microtubule-associated protein 1 light chain 3
LC3-II: Lipidated form of LC3
Nrf2: Nuclear factor (erythroid-derived 2)-like 2
Nqo1: NAD(P)H dehydrogenase, quinone 1
PBS: Phosphate-buffered saline
PM: Polymyositis
ROC: Receiver-operator characteristic
SQSTM1: Sequestosome 1
TBS: Tris-buffered saline
