# Supplementary material for: Activation of the Keap1/Nrf2 stress response pathway in autophagic vacuolar myopathies
Source: Acta Neuropathol Commun. 2016 Oct 31;4:115. doi: 10.1186/s40478-016-0384-6 (PMC5088660; doi:10.1186/s40478-016-0384-6)
Supplement: Additional file 1: Table S1. — Summary of diagnostic criteria used for PM and IBM case classification; adapted from reference [15]. (PDF 11 kb) [file 40478_2016_384_MOESM1_ESM.pdf]

Diagnostic criterion	PM	IBM
Lymphocytic endomysial inflammation[&]	Present	
Inflammatory infiltrate composition[#]	T-cell rich, B-cell poor	
Degenerating / regenerating fibers[&]	Present in random distribution	
Fiber invasion[#]	Present	
Diffuse MHC-1 positivity[#]	Present	
Percentage of COX-negative fibers[&,*]	<1%	Any (usually >1%)
Ragged red fibers[&,*]	Absent	Usually present
Endomysial fibrosis[&]	None or mild	Moderate to severe
Fiber size variation[&]	None or mild	Moderate to severe
Rimmed vacuoles[&]	Absent	Present

Additional file 1: Table S1: Summary of diagnostic criteria used for PM and IBM case classification; adapted from reference [15].

[&] Main criteria (required for diagnosis / classification)
[#] Supporting criteria
* Criteria important for distinguishing classic PM from PM with COX-negative fibers (an IBM precursor condition); see reference [15] for details.
