# Supplementary figures and images for: Activation of the Keap1/Nrf2 stress response pathway in autophagic vacuolar myopathies
Source: Acta Neuropathol Commun. 2016 Oct 31;4:115. doi: 10.1186/s40478-016-0384-6 (PMC5088660; doi:10.1186/s40478-016-0384-6)

Figure S1

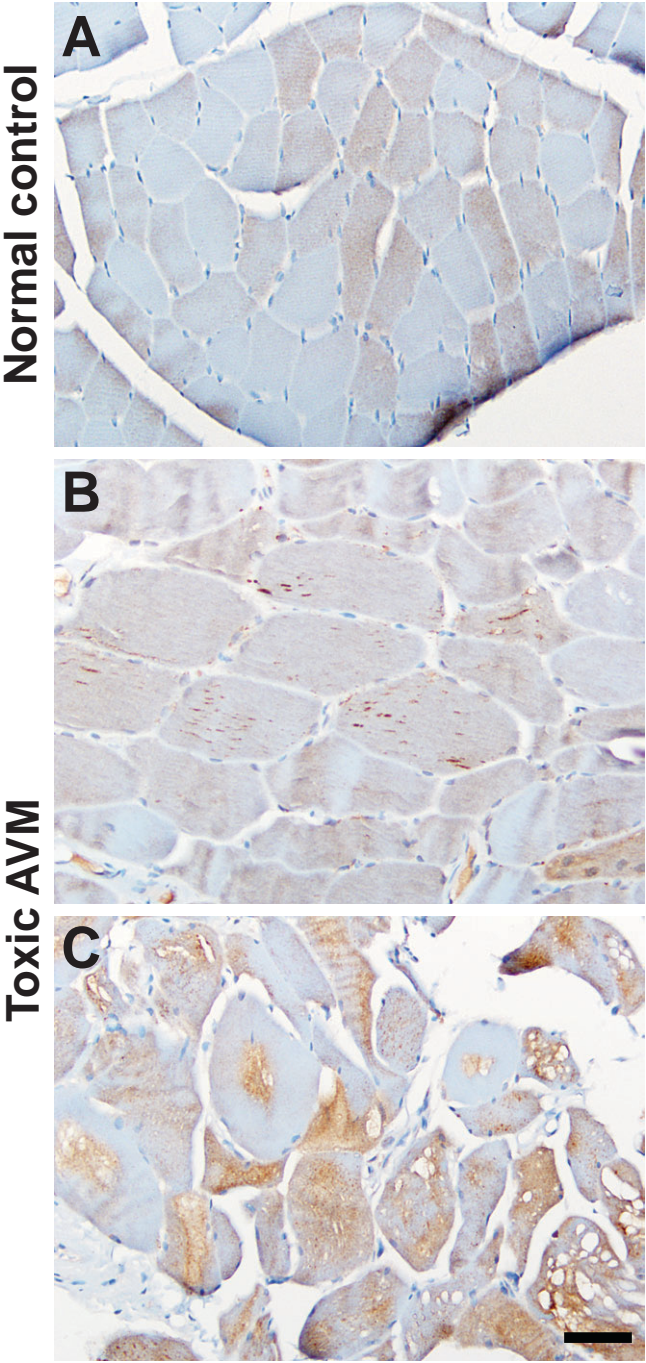

Supplement: Additional file 2: Figure S1. — Keap1 immunohistochemistry with a lower antibody dilution. A. When a lower antibody dilution (1:250) was used for Keap1 immunohistochemistry in the normal skeletal muscle (representative subject #1), diffuse sarcoplasmic staining showed a checkerboard distribution, raising a possibility that Keap1 protein is differentially expressed by slow and fast twitch muscle fibers. B-C. Under these experimental conditions, sequestration of Keap1 into sarcoplasmic puncta was still apparent in the toxic AVM muscle (B, HCQ-treated subject #29; C, colchicine-treated subject #20) but was partially obscured by the background Keap1 staining. Scale bar, 20 μm. (PDF 3954 kb) [file 40478_2016_384_MOESM2_ESM.pdf]
