# Supplementary material for: Activation of the Keap1/Nrf2 stress response pathway in autophagic vacuolar myopathies
Source: Acta Neuropathol Commun. 2016 Oct 31;4:115. doi: 10.1186/s40478-016-0384-6 (PMC5088660; doi:10.1186/s40478-016-0384-6)
Supplement: Additional file 3: Table S2. — mRNA expression level for a subset of the Nrf2-regulated genes (individual subject data; summary graphs are shown in Fig. 6a). (PDF 97 kb) [file 40478_2016_384_MOESM3_ESM.pdf]

Subject ID	Gsr (ng standard)	Nqo1 (ng standard)	G6pd (ng standard)	Hmox1 (ng standard)	Gclm (ng standard)	Gclc (ng standard)
**Normal control group**						
1	0.110	0.595	0.010	4.820	1.698	0.060
2	0.048	0.869	0.006	0.178	0.173	0.000
5	0.025	0.082	0.006	0.295	0.040	0.014
6	0.028	0.143	0.007	0.246	0.107	0.013
7	0.014	0.214	0.003	0.742	1.293	0.010
8	0.003	0.028	0.014	0.345	1.222	0.046
9	0.012	0.114	0.005	0.209	1.163	0.027
**Toxic AVM group**						
22	0.494	2.111	0.059	5.117	0.425	0.080
23	0.066	0.424	0.047	4.460	1.294	0.046
25	0.607	1.468	0.206	1.780	0.097	0.095
27	0.623	42.593	0.110	5.804	2.082	0.080
28	1.199	36.839	0.114	3.711	0.391	0.051
29	0.718	2.860	0.021	0.045	0.142	0.023
30	0.828	3.414	0.192	66.378	11.521	0.024
31	0.630	10.546	0.087	1.006	0.913	0.057
32	0.607	0.438	0.036	5.782	0.124	0.055

Additional file 3:Table S2. mRNA expression level for a subset of the Nrf2-regulated genes (individual subject data; summary graphs are shown in Fig. 6A).
